# Comparative analysis of trueness between conventional and digital impression in dental-supported fixed dental prosthesis with vertical preparation

**DOI:** 10.4317/jced.56967

**Published:** 2020-09-01

**Authors:** Ignacio García-Gil, Celia Perez de la Calle, Carlos Lopez-Suarez, Paula Pontevedra, Maria J. Suarez

**Affiliations:** 1 DDS, MS. Student, Master Program Buccofacial Prostheses and Occlusion, Faculty of Dentistry, University Complutense of Madrid, Madrid, Spain; 2 DDS. Student, Master Program Buccofacial Prostheses and Occlusion, Faculty of Dentistry, University Complutense of Madrid, Madrid, Spain; 3 DDS, MS, PhD. Associate Professor, Department of Conservative Dentistry and Buccofacial Prostheses, Faculty of Dentistry, University Complutense of Madrid, Madrid, Spain; 4 DDS, MS. Researcher, Department of Conservative Dentistry and Buccofacial Prostheses, Faculty of Dentistry, University Complutense of Madrid, Madrid, Spain; 5 MD, Dr Med, DDS, Dr Odont. Professor, Department of Conservative Dentistry and Buccofacial Prostheses, Faculty of Dentistry, University Complutense of Madrid, Madrid, Spain

## Abstract

Biologically oriented preparation technique (BOPT) is a vertical preparation technique without a finish line to create a new anatomic crown with a prosthetic emergence profile. This case report describe the protocol realized digitally in a patient who required a new fixed partial denture (FPD) in the anterior esthetic zone. After time of temporary restoration, definitive conventional (CI) (double-cord retraction and vinyl polysiloxane material), and digital impression (DI) with three different intraoral scanner (IOS) (Trios®, True Definition® and iTero®) were taken. All digital impression were obtained through three different scans: temporary restoration in the mouth after healing period, prepared teeth, and temporary restoration out of the mouth. To establish which of the IOS was the most accurate, it was necessary to compare the STL files obtained from each of the IOS with the STL file of the conventional impression, which was digitized with a laboratory scanner (3Shape D800). All these STL were imported to a software (ExoCAD 2.4 Plovdiv®), and they were superimposed. To establish the difference in trueness with SC, 6 points were chosen, 3 points in teeth, and another 3 points in soft tissue. The mean measurement in terms of trueness in teeth were: STS (0,039 mm), SI (0,054 mm), STD (0,067 mm); and in soft tissue were: STS (0,051 mm), SI (0,09 mm), STD [0,236 mm]. The IOSs showed differences between them in terms of trueness, being the Trios the most accuracy IOS. Final restoration was fabricated and cemented. The patient was examined at 3, 6 and 12 months, without any type of biological or mechanical complications. Digital impression with an IOS seems to be a viable alternative to perform zirconia FPD in the BOPT tecbique.

** Key words:**Intraoral scanners, accuracy, vertical preparation, precision, CAD-CAM, prosthodontics.

## Introduction

One of the main complications and a long-term challenge in the field of fixed dental prosthesis is the appearance of gingival recessions (1). The etiology of this event is multifactorial (chronic inflammation due to poor adjustment of the restoration, trauma, gingival biotype, or inadequate dental preparation), and it may be lead to both esthetic and biological complications (2). In order to minimize the appearance of all these complications, Loi and Di Felice described a protocol of tooth preparation and management of provisional restoration to create a new anatomic crown with a prosthetic emergence profile that simulates the shape of the natural tooth (3). This protocol was named biologically oriented preparation technique (BOPT), and consists in stabilizing the clot formed eliminating the cementoenamel junction (CEJ) with a rotary instrument penetrating the gingival sulcus, with a temporary crown, and adapting it to the gingival margin. Once tissue healing time has expired, an either conventional or digital definitive impression must be carried out (4).

Traditionally, the treatment approach in the field of fixed prosthodontics which ensured the best results, has consisted in a conventional impression technique with different materials (polyether or polyvinyl siloxane), to make the stone casts, and to fabricate a porcelain-fused-to-metal restoration. This protocol of treatment is still considered the standard clinical reference for replicating the intraoral situation (5).

Subsequently, the introduction of Computer Aided Design/Computer Aided Manufacturing (CAD/CAM) technology caused an improvement in precision and reproducible production results (6). The implication of these new technologies in dentistry, has caused the appearance of intraoral scanners (IOS), wich are being used for digital impression (DI). The intraoral scanning was developed to solve different obstacles and challenges for both patient and dentist, such as: nausea, unsatisfactory taste, discomfort, time consumption, and better cost-benefit ratio (7). For these reasons, a paradigm shift is occurring in the field of impression technique in fixed prosthodontics. Despite of these potential advantages, several factors that can affect to the result of scanning must be addressed such as: patient and operator movement, presence of saliva and/or blood, obstructions by tongue and/or cheek, space reduced to IOS, and reflection of light by intraoral structures (8). Another important factor is the mathematical quality of the files obtained from the IOS, which is influenced by the accuracy and resolution (9).

The accuracy of IOS is very important, because an accurate impression is the first step to guarantee the passive fit of the final restoration. Accuracy can be defined as the sum of the trueness and the precision. Trueness, is defined as “closeness of agreement between the expectation of a test result or a measurement result and a true value”; whereas precision can be considered the “closeness of agreement between indications or measured quantity values obtained by replicate measurements on the same objects under specified conditions”. In other words, an excellent IOS should be able to obtain scanning with high trueness, less deviation of the referenced object; and high precision, more reproducible measurements (10). Finally, the resolution of IOS is another key factor which can affect to result of scanning, since directly influences in the visualization of details such as the preparation line. Resolution is determined by the density and number of triangles that constitutes the mesh of the scanning.

Focusing on the accuracy of IOS in the field of prosthodontics, the aim of this clinical study was to establish the trueness of conventional and digital impression in dental-supported fixed prosthesis using the BOPT technique.

## Case Report

A 75-year-old female, was referred to the Department of Conservative Dentistry and Buccofacial Prostheses (Faculty of Dentistry, Complutense University of Madrid, Madrid, Spain) to replace an ancient porcelain-fused-to-metal dental-supported fixed partial denture (FPD) in position #25 (second upper left premolar) with medial cantilever to cover position #24 (first upper left premolar). The reasons to replace the FPD were the rejection of using dental implants and esthetic demand. The patient’s medical history included hypertension, treated with nifedipine (10 mg 1-0-0). The patient had excellent oral hygiene with no unhealthy habits, and no other significant medical conditions were reported. Before treatment, patient was informed of the study objective and clinical procedures, and was asked to provide written informed consent.

Before dental preparation, complete arch conventional impressions were made with an irreversible hydocolloid material (Cavex Impressional, Cavex, Haarlem, Netherlands), and were sent to the dental lab to obtain type IV plaster casts (GC Fujirock EP OptiXscan, GC Europe, Leuven, Belgium), and fabricate the temporary FPD.

**Figure 1:**
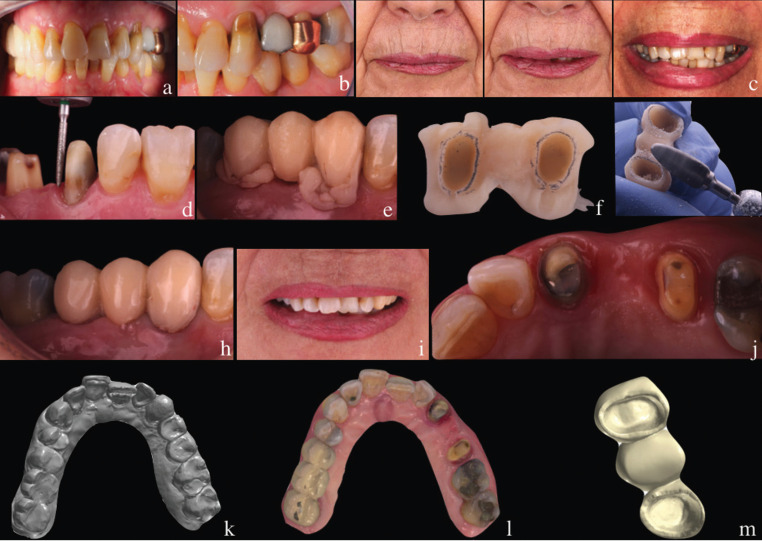
A) Preoperative intraoral view in frontal vision; B) Preoperative intraoral view in lateral vision; C) Sealing, rest and maximum smile; D) Teeth prepared without finish line; E) Relined of temporary FPD; F) Modification of temporary FPD to obtain ideal gingival emergence, G) Polished of temporary FPD; H) Cementation of temporary FPD; I) maximum smile with temporary FDP; J) soft tissue state after provisional time; K) Digital impression of Temporary FPD cemented; L) Digital impression of abutment teeth; M) Extraoral scanning of the temporary FPD.

Afterwards, the porcelain-fused-to-metal FPD was removed, and the upper left canine and second premolar were prepared with the BOPT technique, eliminating the horizontal finish line with a conical bur (862.534.012, BOPT drills; Sweden & Martina, Due Carrare, Italy)] tilted obliquely 15 degrees to the tooth´s long axis, to eliminate the preexisting prepared finish line, respecting biological width. The provisional hollowed acrylic FPD, was relined with cure metacrylate (RCB-KIT. Sweden & Martina), and adapted to the new emergence of the tooth’s crown created (3). After four weeks of healing process and changes to achieve gingival adaptation, definitive conventional (CI) and digital impression (DI) with three different IOS [Trios® (3Shape, Copenhagen, Denmark); True Definition® (3M ESPE, St Paul, USA); iTero® Element 2 (Align Tecnology, INC, San Jose, USA)] were made (Fig. 1). Both, definitive CI and DI were made by the same operator,the same day and with the same conditions (temperature, time, humidity), to guarantee the smallest appearance of bias between the different impressions.

-Definitive Conventional Impression (CI), was made with double impression technique using double-cord retraction (Ultrapack; Ultradent Products Inc, Köln, Germany) and vinyl polysiloxane material (Aquasil Ultra Monophase/LV; Dentsply Sirona, York, USA). This impression was scanned by a laboratory scanner (3Shape D/R800; 3Shape, Copenhagen, Denmark)), with an accuracy < 15 μm as provided by the manufacturer. The objective of not pouring this impression was to produce a STL based on negative of the impression (SC), without introducing cumulative errors caused by an additional step.

-Definitive Digital Impressions (DI) were made by the following clinical protocol (11). A first scan with the provisional restoration in the mouth, a second scan of the dental preparations, and a third scan of the provisional restoration out of mouth. The second and the third scans are very important, because they supply information about the gingival sulcus, where the dental technician will determine the end of the final restoration. Best fit of each of these three STL were aligned to create and to design the final restoration in a software (3Shape Implant Studio, 3Shape). These STL were obtained from the three different IOS: Trios® (STS); True Definition® (STD); iTero® (SI).

**Figure 2:**
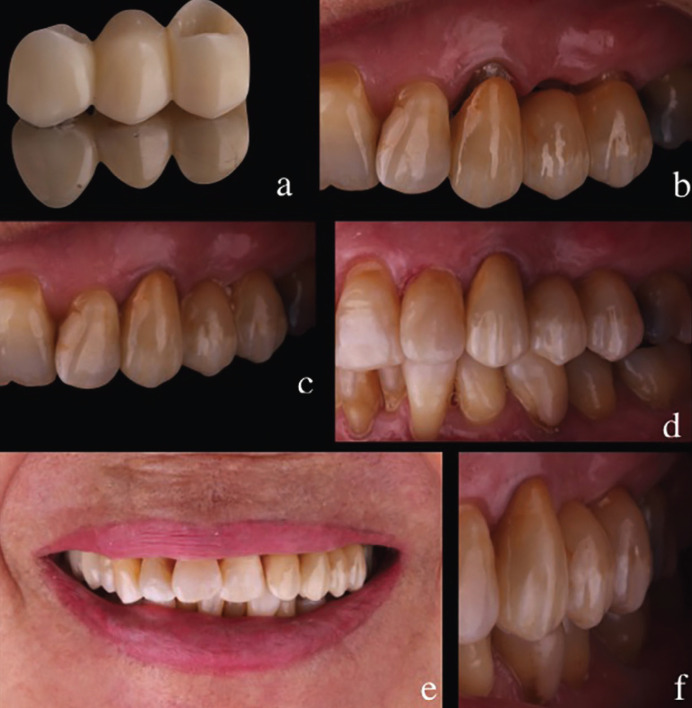
A) PolyMethylMethAcrylate (PMMA) test; B) Try-in previous cementation; C) Final restoration; D) 12 months follow-up; E) Smile after 12 months; F) Soft tissue state after 12 months.

Once the impressions were made, a PolyMethylMethAcrylate (PMMA) test was designed and printed in the dental laboratory to be tried in the patient. The occlusion was adjusted, following the appropriate procedures, and the marginal adjustment was verified. Finally, the FPD was made using the IPS e.max ZirPress LT system (Ivoclar Vivadent, Schaan, Liechtenstein), and cemented with an auto-polymerizing resin cement (RelyX Unicem 2 Automix; 3M ESPE, Seefeld, Germany). The occlusion was tested again, and instructions in oral hygiene, and care of the new FPD were given to guarantee the success of the restoration. The patient was examined at 3, 6 and 12 months, and no biological or mechanical complications were observed (Fig. 2).

**Figure 3:**
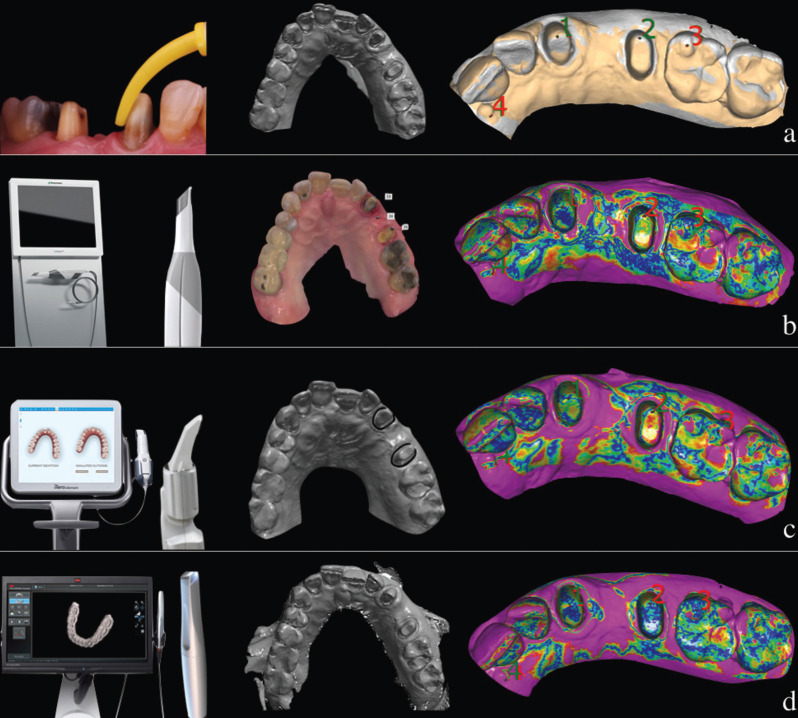
A) Conventional Impression and STL (SC); B) DI with Trios, STS, BestFit ; C) DI with iTero, SI, BestFit ; D) DI with True Definition, STD, BestFit.

With the aim to evaluate the trueness of different DI compared to test impression (SC), all of the scans obtained were subsequently imported and cut into an engineering software (ExoCAD 2.4 Plovdiv; ExoCAD GmbH, Darmstadt, Germany), using a preconFigured cutting tool (in order to reproduce the same cuts). STL file obtained of CI was named as scanning test (SC), which was used as a STL reference to perform different Best fit alignments between the different scannings of IOSs. In this way, it was possible to evaluate the precision changes of each of the STL obtained from the three IOS with conventional impression. Furthermore, to perform the different measurements the following points were established: 3 points in teeth (#23, #25 and #26), and other 3 points in soft tissue (interdental papilla, distal papilla of #2.3 and mesial papilla of #25), (Fig. 3).

Summary of statistics for all three scanners and conventional impression for substrate trueness are presented in Table 1. The mean measurements in terms of trueness in teeth were as follow: STS [0,039 mm], SI [0,054 mm], STD [0,067 mm]; while in soft tissue were as follow: STS [0,051mm], SI [0,09 mm], STD [0,236 mm]. The IOSs showed differences between them in terms of trueness, being Trios IOS the most accuracy, and True Definition IOS the one with the worst results.

**Table 1:**
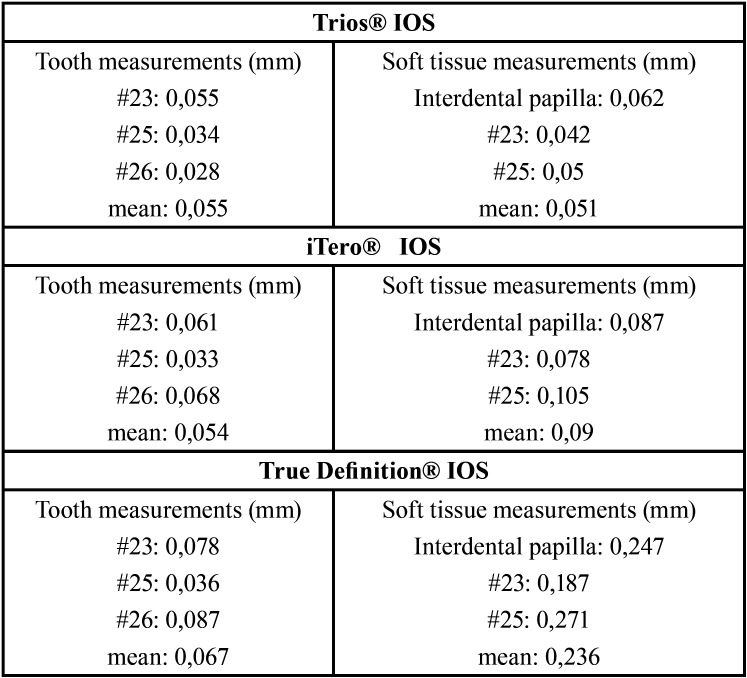
Trueness measurements of STL obtained with intraoral scanner compared to conventional impression.

## Discussion

One of the most challenge in the field of prosthodontics had been the incorporation of digital technology to carry out any type of dental procedure. First great change in this field was the introduction of CAD/CAM-technology which allowed the production of monolithic fixed dental prostheses of different materials, which it is a great help to any dental technician or clinician. Moreover, other new tools have been developed to obtain best results as milling or 3D–printing, rapid prototyping procedures with secondary computer-assisted production, or the introduction of IOS (12). IOS offers a multiple advantages compared to conventional impression, specially in terms of patient’s comfort and cost-benefit ratio (7,13). For these reasons, several *in vivo* or *in vitro* studies that analyze this technology are appearing (14).

The majority of IOSs studies analyze the accuracy or trueness of these systems among them, or compared to conventional system. The reason is to try to demonstrate the effectivity of IOS, since this is one of the most important factor in prosthodontics to long-term success, and it can be reduced the number of clinical steps (15).

However, up to date there is no clinical study or case report which analyze the accuracy of CI and DI in a real case treated with the BOPT technique digitally. The study results revealed important information, because it was determined the employ of IOS to perform the BOPT technique digitally for dental supported prosthesis with accepTable values, and no relevant differences were found compared to conventional impression.

Thus, digital impression with IOS may be a very attractive alternative, since it offers many advantages over conventional impressions, and a digital complete workflow is allowed. Differences between different IOS were found in terms of trueness, and could be due to multiple reasons such us the type and technolgy of the IOS, the experience of the operator, or the presence of saliva and/or blood. Nevertheless, more clinical and long-term studies are needed to establish the accuracy of the IOSs in the BOPT tecnique.

## Conclusions

Within the limitations of the study, CI or DI can produce final dental-supported restoration with the BOPT tecnique showing accepTable results.
